# Livingstone College

**Published:** 1904-06-18

**Authors:** 


### LIVINGSTONE COLLEGE.

### COMMEMORATION DAY PROCEEDINGS, JUNE 9th, 1904.

At the Commemoration Day proceedings at Livingstone College on June 9th, about 200 visitors were present at the meeting in the garden, including, among others, Bishop Cassells, Mr. Frank Wilson, Dr. Livingstone's son-in-law, and Captain Forbes, upon whose ship Dr. Livingstone had travelled.

The Principal, Dr. C. F. Harford, made a statement concerning the progress of the College. He referred to the brilliant advances which had been made during the past year in the department of research in tropical medicine, which offered a steadily growing argument in favour of the work of Livingstone College. He contended that the College played a humble but important part in the work of hygienic and sanitary reform, both by the missionaries trained within its walls—who are now scattered all over the world—and by the publications of the College which circulate widely among European residents in tropical climates; and he specially drew attention to the new Livingstone College Year Book, published that day, containing proofs of the value of the training received, from students in all parts of the world, and also including special hints on the preservation of health in tropical climates, published for the first time in this Year Book. Livingstone College is endeavouring by every possible means to keep step with the progress of modern science by the purchase of suitable apparatus, and by the equipment of a small laboratory, the opening of which by Professor G. Sims Woodhead, the chairman of the meeting, took place on Commemoration Day.

Changes had been made in the course of training during the past year so as to admit students for shorter periods of training than the full nine months, which is the length of the regular session. As yet many of the missionary societies did not realise the importance of medical training for their missionaries, but most of them were now giving special attention to the matter and hoped to make much more use in the future of Livingstone College.

The Treasurer, Mr. R. L. Barclay, gave a statement with regard to the financial position of the College. He referred to the gift of £4,000 which had been made during the past year by the Principal and Mrs. C. F. Harford, part of which was being devoted to taking over the mortgage on the property, the fund being placed in the hands of the Trustees, and part being used for the equipment of the new laboratory and the purchase of apparatus, whilst it was hoped that a certain sum could also be used for the provision of better means of recreation for the students. It would be impossible, however, for this improvement to be carried out unless considerable donations—to the amount at least of £600—were made to prevent a deficiency on the year's accounts. The Committee earnestly hoped that this might be forthcoming, as they did not think the gift of the Principal should be devoted to current expenses, but rather should be applied to the purchase of apparatus, or the making of other improvements which would permanently benefit the College.

Professor Sims Woodhead, who presided, referred to the way in which Livingstone had revolutionised history, and thought it was very appropriate that the College should be called by his name, as he had himself realised the immense importance of medical knowledge in his work as a missionary. He (the Professor) held that it was the duty of every society to see to it that no one goes to the mission field who is not in some way prepared, at any rate by a short course of study, to take care of his or her own bodily health, and the health of those dependent upon them; and he felt that the various missionary societies of this and other countries must be brought to feel that they have a very grave responsibility in this matter.

After referring to the death of Sir Henry Stanley, who had been closely associated with Livingstone's life and work, he quoted a saying of Dr. Livingstone's, to the effect that fever was the chief obstacle to the opening up of Africa, and it was his earnest desire that this should be removed in order that Africa might be opened up to Christianity and civilisation. The Professor continuing, referred in cordial terms to the equipping of the new laboratory, which he declared open. He believed that it would be a great help to missionaries to have demonstrations showing them the micro-organisms which are the causes of disease, so that they may be able more easily to prevent them.

In conclusion he appealed to the missionary societies to join together in supporting Livingstone College. He suggested that each society should make a certain contribution each year to the College, and for this sum have the privilege of sending a certain number of students. He believed that this would be a splendid way of spending the funds of the society, and amply repay them by the results which would be obtained.

Three interesting testimonies were given as to the value of medical work in the mission field, by Mr. William Pettigrew, of the American Baptist Missionary Union, from Assam; Mr. Bond, of the Wesleyan Missionary Society, from West Africa; and Mr. E. Dennis, of the Church Missionary Society, from the River Niger.

Dr. William Wilson spoke as one who had been a missionary in Madagascar and also the Secretary of the Friends' Foreign Missionary Society. He believed that even a little medical knowledge was of enormous value in dealing with the natives. It was also of the greatest importance for the sake of the missionaries themselves, who often have to meet great grave risks, and do so without the slightest fear. He was of the opinion that a little knowledge of the means of preservation of the health would have saved many lives and averted a great deal of sickness. Sometimes when missionaries came home invalided they were accused of carelessness, but he believed that the blame lay chiefly with the missionary society who had sent them out without proper equipment as regards their health. He hoped that those present would make practical use of the afternoon's meeting by impressing upon the missionary boards and committees the extreme importance of equipping their missionaries with some medical knowledge. He considered that the nine months' course was all too short, and that missionaries should have the best training in this direction that it was possible to obtain.

Mr. Frank Wilson offered the concluding prayer and Bishop Cassells pronounced the Benediction.

The guests afterwards had tea, inspected the College and the laboratory, and a demonstration with the new electric lantern concluded a most successful day.

